# Biomarkers in Natural Fish Populations Indicate Adverse Biological Effects of Offshore Oil Production

**DOI:** 10.1371/journal.pone.0019735

**Published:** 2011-05-23

**Authors:** Lennart Balk, Ketil Hylland, Tomas Hansson, Marc H. G. Berntssen, Jonny Beyer, Grete Jonsson, Alf Melbye, Merete Grung, Bente E. Torstensen, Jan Fredrik Børseth, Halldora Skarphedinsdottir, Jarle Klungsøyr

**Affiliations:** 1 Department of Applied Environmental Science (ITM), Stockholm University, Stockholm, Sweden; 2 Department of Biology, University of Oslo, Oslo, Norway; 3 Norwegian Institute for Water Research (NIVA), Oslo, Norway; 4 National Institute of Nutrition and Seafood Research (NIFES), Bergen, Norway; 5 International Research Institute of Stavanger (IRIS), Stavanger, Norway; 6 Department of Mathematics and Natural Science, University of Stavanger, Stavanger, Norway; 7 Department of Medical Biochemistry, Stavanger University Hospital, Stavanger, Norway; 8 Marine Environmental Technology, SINTEF Materials and Chemistry, Trondheim, Norway; 9 Institute of Marine Research (IMR), Bergen, Norway; Institute of Marine Research, Norway

## Abstract

**Background:**

Despite the growing awareness of the necessity of a sustainable development, the global economy continues to depend largely on the consumption of non-renewable energy resources. One such energy resource is fossil oil extracted from the seabed at offshore oil platforms. This type of oil production causes continuous environmental pollution from drilling waste, discharge of large amounts of produced water, and accidental spills.

**Methods and principal findings:**

Samples from natural populations of haddock (*Melanogrammus aeglefinus*) and Atlantic cod (*Gadus morhua*) in two North Sea areas with extensive oil production were investigated. Exposure to and uptake of polycyclic aromatic hydrocarbons (PAHs) were demonstrated, and biomarker analyses revealed adverse biological effects, including induction of biotransformation enzymes, oxidative stress, altered fatty acid composition, and genotoxicity. Genotoxicity was reflected by a hepatic DNA adduct pattern typical for exposure to a mixture of PAHs. Control material was collected from a North Sea area without oil production and from remote Icelandic waters. The difference between the two control areas indicates significant background pollution in the North Sea.

**Conclusion:**

It is most remarkable to obtain biomarker responses in natural fish populations in the open sea that are similar to the biomarker responses in fish from highly polluted areas close to a point source. Risk assessment of various threats to the marine fish populations in the North Sea, such as overfishing, global warming, and eutrophication, should also take into account the ecologically relevant impact of offshore oil production.

## Introduction

The offshore oil industry discharges produced water into the sea, both at the surface and at various depths [1], [2]. The two primary sources of produced water are fossil water present in the reservoir and seawater injected into the reservoir to maintain pressure. Produced water contains a complex mixture of organic and inorganic substances with large variations in the amount and composition between reservoirs and over the lifetime of a single reservoir. In the Oslo-Paris (OSPAR) Convention area, the discharge of oil via produced water is regulated on the basis of total hydrocarbon concentration rather than volume or toxicity. At the time of this study the maximum allowed concentration was 40 mg/L. Procedures generally used to limit the petroleum pollution include separation of the hydrocarbons prior to discharge and, less frequently, reinjection of the produced water into the reservoir.

At the time of this study, estimates of the annual worldwide release of oil in the form of produced water into the oceans ranged between 19,000 and 62,000 tons [2]. The corresponding estimate for the North Sea was a total of approximately 8,200 tons according to estimations from Norway, Denmark, the Netherlands, and the United Kingdom [2]. The discharge is steadily increasing, since the volumes of produced water increase when oil reserves become depleted. In 1992, the discharge of oil-based drilling fluids, also known as muds, to the Norwegian continental shelf was banned. These muds are still used, but must now be reinjected into the reservoir or brought to shore for cleaning and storage. Hence, in recent years muds released directly into the sea have been primarily water-based.

**Figure 1:**
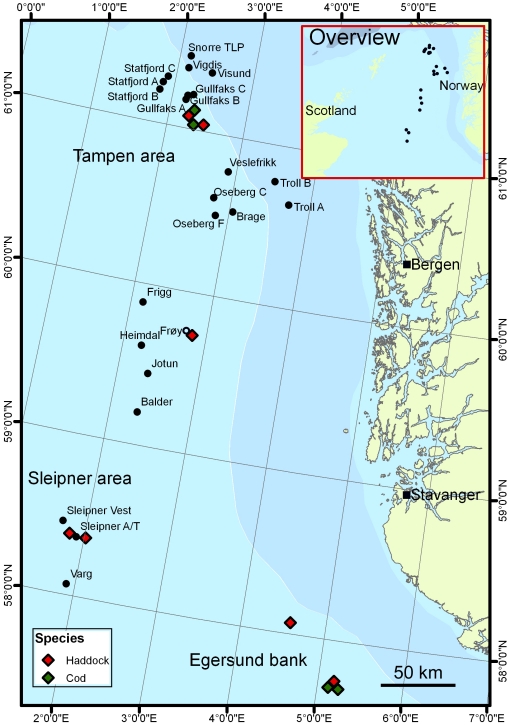
The investigated areas in the North Sea. Black dots indicate active oil platforms. The black ring at Frøy indicates a former oil production site, which has been inactive since 2001, and from which the platform was removed in 2002. Diamonds indicate the sites where haddock (*Melanogrammus aeglefinus*) and Atlantic cod (*Gadus morhua*) were collected.

We have investigated two areas in the North Sea with extensive oil production: the Tampen area and the Sleipner area (Fig. 1). The daily discharge of produced water in these two areas, as documented by the oil industry in 2002, was 278,000 m^3^ and 10,500 m^3^, respectively. The value for Tampen represents an approximately 10-fold increase in the discharge of produced water in that area over a decade [1]. At the time of this study, the hydrocarbon content of the produced water released in the North Sea by the offshore oil industry varied between 15 and 40 mg/L [2]. These figures give an estimate of the daily release of oil and grease of 4–11 tons at Tampen and 0.1–0.4 tons at Sleipner. Accordingly, the discharge may differ by a factor of ten or more between the two areas. The Egersund bank, with no oil or gas production, was used as control. Some preliminary results from this study were reported by Hylland et al. 2006 [3].

Genotoxicity was investigated also at the former oil production site Frøy, which has been inactive since 2001 and from which the platform was removed in 2002. The only visible traces of the previous activities at this site were an approximately 50×100 m pile of drill cuttings and several buried pipelines on the sea floor. In order to ensure a correct interpretation of the DNA adduct and ethoxyresorufin *O-*deethylase (EROD) activity data, control material was also collected southwest of Iceland, i.e. from remote waters without any known point sources of pollution.

The aim of this study was to investigate if fish health is affected by oil production in the North Sea. The responses of a battery of established biomarkers proved to be similar to the biomarker responses in fish from highly polluted areas close to a point source. The results also indicate significant background pollution in the North Sea.

## Materials and Methods

### Fish

Haddock (*Melanogrammus aeglefinus*) was collected in the Tampen area, the Sleipner area, and at the Egersund bank in the autumn 2002 (Fig. 1), at Frøy in the summer 2003 (Fig. 1), and southwest of Iceland in the spring 2004. Atlantic cod (*Gadus morhua*) was collected in the Tampen area and at the Egersund bank in the autumn 2002 (Fig. 1). Within each species, the material was homogeneous with respect to length, weight, gonad and liver size, and there were no differences between the sexes in the investigated variables. In accordance with contemporary regulations, no ethical permit was needed for the collection and killing of the fish.

### Sampling

The fish were caught by trawling and maintained in basins during sampling in on-board laboratories. The fish were killed with a hard blow on the head, and the abdomen was opened. The gall bladder was dissected without contaminating other inner organs and the bile was collected in cryotubes. The liver was dissected and pieces were put in cryotubes. The liver piece for analysis of DNA adducts was taken from the central part of the liver. Epaxial muscle pieces were dissected and put in cryotubes. All cryotubes were immediately snap frozen in liquid nitrogen. The samples were later stored at −80°C until further preparation and/or analysis.

### Homogenisation of liver samples

The liver samples were thawed on ice and homogenised in 5 volumes of ice-cold 0.1 M phosphate buffer, pH 7.8, containing 0.15 M KCl, 5% glycerol, and 1 mM dithiothreitol, using a 9 mL Potter-Elvehjem homogeniser (size 21) with four up and down strokes at 400 revolutions per min and under constant cooling with ice. The homogenate was centrifuged at 10,000 g and 4°C for 30 min. The supernatant (S9 fraction) was collected and centrifuged at 50,000 g and 4°C for 2 h. The supernatant (cytosolic fraction) was collected, mixed and divided into aliquots, which were put in cryotubes. The pellet (microsomal fraction) was resuspended in the same medium as used for the homogenisation and divided into aliquots, which were put in cryotubes. All cryotubes were immediately snap frozen in liquid nitrogen. The samples were later stored at −80°C until analysis.

### Bile metabolites of PAHs

The bile samples were diluted 1∶1600 with a mixture of methanol and water (1∶1), and metabolites of polycyclic aromatic hydrocarbons (PAHs) were semi-quantified with fixed wavelength fluorescence (FF) spectrometry. Fluorescence was measured at the excitation and emission wavelength pairs 290/335 nm (2- and 3-rings), 341/383 nm (4-rings), and 380/430 nm (5- and 6-rings), optimised for the detection of metabolites of naphthalene, pyrene, and benzo[a]pyrene, respectively, and with a slit width of 2.5 nm for all excitation and emission wavelengths [4]. The results were expressed as µg pyrene fluorescence equivalents (PFE) per mL bile. In addition to this, the identity of the measured PAH metabolites was confirmed with synchronous fluorescence spectrometry with a difference of 42 nm between the excitation and emission wavelengths [5].

Analysis of specific PAH metabolites in the bile (i.a. hydroxylated forms of naphthalene, phenanthrene, and pyrene) was performed by gas chromatography/mass spectrometry (GC/MS). These metabolites were first deconjugated by incubation with β-glucuronidase (200 U/µL bile) containing sulfatase activity (10 U/µL bile) in 0.4 M sodium acetate buffer, pH 5.0, at 40°C for 2 h [6]. Hydrolysed metabolites were extracted with ethyl acetate (3 times with 0.5 mL each time), dried over anhydrous sodium sulphate, and concentrated to a volume of 0.5 mL. Trimethylsilyl ethers of the deconjugated metabolites were prepared by the addition of 0.2 mL bis(trimethylsilyl)trifluoroacetamide and incubation of the samples at 60°C for 2 h. Standards, instrument settings, and additional treatment of samples, including the analysis of certified reference materials, have been described previously [6]. Most PAH metabolites gave only a trace signal, whereas the concentration of 2-hydroxynaphthalene was possible to quantify. The results were expressed as ng 2-hydroxynaphthalene per mL bile.

### Hepatic enzyme activities

Microsomal cytochrome P4501A (CYP1A) was measured both fluorometrically, on the basis of its ethoxyresorufin *O-*deethylase (EROD) activity [7], and by western blotting, using standard procedures described previously [8]. Haddock CYP1A was blotted with a polyclonal anti-fish CYP1A antiserum (CP226, Biosense, Bergen, Norway), whereas Atlantic cod CYP1A was blotted with a monoclonal anti-cod CYP1A antiserum (NP-7, Biosense, Bergen, Norway). Cytosolic glutathione transferase activity was measured with 1-chloro-2,4-dinitrobenzene as the second substrate [9]. Cytosolic glutathione reductase was measured as the oxidation of reduced nicotinamide adenine dinucleotide phosphate (NADPH) [10]. Cytosolic selenium-dependent glutathione peroxidase activity was measured with hydrogen peroxide as the substrate and sodium azide as a catalase inhibitor [11]. Protein was quantified with the method described by Lowry et al. 1951 [12]. The method was adapted for plate-reader, and bovine immunoglobulin was used as the standard. Specific enzyme activities were expressed as the amount of substrate converted per min and mg protein. The amount of CYP1A enzyme was expressed as absorbance per mg protein.

### Fatty acids

The total lipid in epaxial muscle and in the liver was extracted with a mixture of chloroform and methanol (2∶1, 4 mL per 0.2 g sample). The methyl ester of the fatty acid 19∶0 was added as an internal standard. The samples were stored at −20°C overnight and then filtered and saponified. The fatty acids were esterified in methanol containing 12% BF_3_. The resulting fatty acid methyl esters were separated, as described previously [13], and identified on the basis of their retention times, which were determined with a standard mixture of fatty acid methyl esters (Nu-Check-Prep, Elysian, MN, USA). The following fatty acids were measured: 18:3n-3, 20:4n-3, 20:5n-3, 22:5n-3, 22:6n-3, 18:2n-6, 20:2n-6, and 20:4n-6. The ratio of n-3 to n-6 fatty acids in the muscle was computed. The concentration of arachidonic acid (20:4n-6) in the liver was expressed as µg arachidonic acid per g tissue (wet weight).

### Muscle α-tocopherol

Epaxial muscle tissue was saponified in a saturated solution of pyrogallol, ascorbic acid, and ethylenediaminetetraacetic acid (EDTA) in 80% aqueous ethanol containing 20 mM KOH at 100°C for 20 min. α-Tocopherol was extracted with hexane, separated with normal-phase high performance liquid chromatography (HPLC), and quantified by its fluorescence [14]. The excitation wavelength was 289 nm and the emission wavelength was 331 nm. The concentration of α-tocopherol in the muscle was expressed as µg α-tocopherol per g tissue (wet weight).

### Hepatic DNA adducts

The liver samples were semi-thawed and the DNA extracted and purified as described by Dunn et al. 1987 [15], Beach and Gupta 1992 [16], and Reichert and French 1994 [17] with slight modifications described by Ericson et al. 1998 [18] and Ericson and Balk 2000 [19]. The DNA adducts were enriched with the nuclease P1 method: incubation for 45 min with 0.8 µg nuclease P1 per µg DNA [16], [20]. The DNA adducts were then radio-labelled with 5′-[γ-^32^P]-adenosine triphosphate ([γ-^32^P]-ATP) in a reaction catalysed by T_4_-polynucleotide kinase. The radio-labelled DNA adducts were separated and cleaned-up by multidirectional thin-layer chromatography (TLC) on polyethyleneimine cellulose sheets. The sheets were produced by us in order to obtain maximum resolution of DNA adducts formed by large hydrophobic aromatic xenobiotics, such as PAHs with 4, 5, or 6 rings [17], [19], or other correspondingly large hydrophobic xenobiotics. Finally, the DNA adducts were visualised and quantified by storage phosphor imaging with a PhosphorImager™ and ImageQuant 5.0 software. The frequency of DNA adducts was expressed as nmol adducts per mol normal nucleotides. Positive and negative controls were processed parallel with the samples and confirmed the reliability of the assay [21].

### Statistics

The data (Dataset S1) are presented as means ±95% confidence intervals. Differences between station means were determined by analysis of variance (ANOVA) followed by the post-hoc test Fisher's protected least significant difference (Fisher's PLSD). A *p*-value <0.05 was considered significant. Two-way ANOVA with station and sex as factors showed that there were no differences between the sexes in the investigated variables. The software Statview 5.0 (SAS Institute Inc., Cary, NC, USA) was used for the analyses.

## Results and Discussion

### Evidence of PAH exposure and uptake

**Figure 2:**
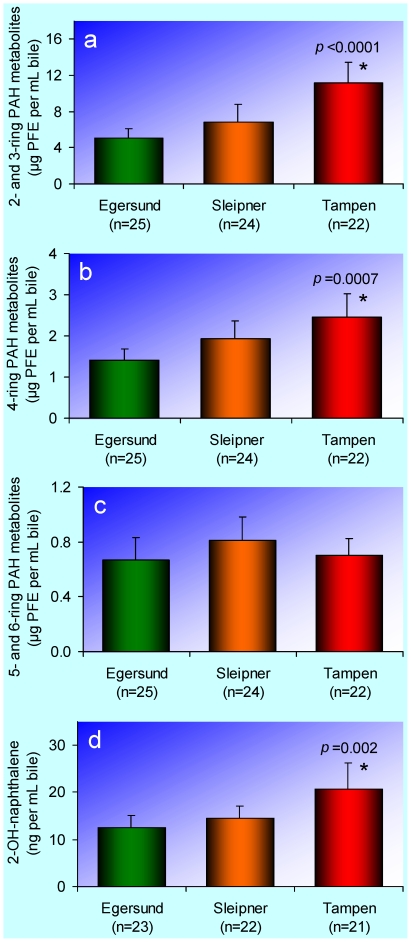
Metabolites of polycyclic aromatic hydrocarbons (PAHs) in the bile. In **a–c** bile metabolites in haddock (*Melanogrammus aeglefinus*) were measured as pyrene fluorescence equivalents (PFE). **a**, 2- and 3-ring PAH metabolites. **b**, 4-ring PAH metabolites. **c**, 5- and 6-ring PAH metabolites. **d**, Enzymatically deconjugated 2-hydroxynaphthalene in haddock. The bars represent arithmetic means and the error bars 95% confidence intervals. An asterisk (*) indicates that the mean is significantly different (*p*<0.05) from the mean for the Egersund bank (control).

The presence of PAH metabolites in the bile is evidence of PAH exposure and uptake. In fish, as in other vertebrates, PAHs exert much of their toxicity as reactive intermediate metabolites formed during the enzymatic biotransformation into more water-soluble compounds destined for excretion [22]–[24]. Consequently, the presence of PAH metabolites in the bile is also indicative of adverse intracellular toxic effects in the rest of the body [25]. The most toxic PAHs in produced water have not been identified. Therefore, we analysed the bile with respect to unspecific PAH metabolites of three different sizes: 2- and 3-rings, 4-rings, and 5- and 6-rings (Fig. 2a–c). We also analysed the specific bile PAH metabolite 2-hydroxynaphthalene (Fig. 2d), since naphthalene is a major PAH in produced water [1]. In haddock from Tampen, we found elevated concentrations of bile PAH metabolites with 2, 3, and 4 rings, as well as the specific bile PAH metabolite 2-hydroxynaphthalene, and there was a graded response in these variables with respect to the intensity of the oil production in the investigated areas (Fig. 2a,b,d). No corresponding pattern was obtained for PAH metabolites with 5 and 6 rings (Fig. 2c). Other recent studies confirm the utility of bile metabolites in fish as markers of exposure to produced water [26], [27].

### Induction of hepatic enzyme activities

The cytochrome P450 system catalyses the first biotransformation step in the conversion of PAHs and other xenobiotics into more water-soluble compounds. The cytochrome P450 isoform 1A (CYP1A) is up-regulated by PAH exposure in a process called substrate induction. CYP1A catalyses many reactions *in vivo*, and its activity may be measured *in vitro* as the deethylation of the artificial substrate ethoxyresorufin. This activity is commonly referred to as EROD activity. The EROD activity is often assumed to be directly proportional to the amount of active CYP1A enzyme. In this study, we measured both the EROD activity and the amount of CYP1A enzyme in the liver in order to substantiate the extent of substrate induction of CYP1A.

**Figure 3:**
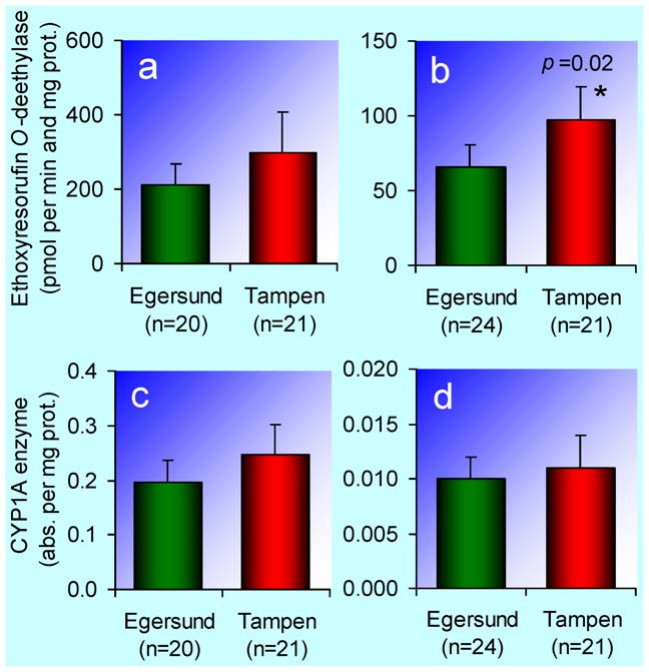
Hepatic ethoxyresorufin *O*-deethylase (EROD) activity and amount of hepatic cytochrome P4501A (CYP1A) enzyme. **a**, EROD activity in haddock (*Melanogrammus aeglefinus*). **b**, EROD activity in Atlantic cod (*Gadus morhua*). **c**, CYP1A enzyme in haddock. **d**, CYP1A enzyme in Atlantic cod. The bars represent arithmetic means and the error bars 95% confidence intervals. An asterisk (*) indicates that the mean is significantly different (*p*<0.05) from the mean for the Egersund bank (control).

The EROD activity was elevated in Atlantic cod from Tampen (Fig. 3b), and there were tendencies towards elevated EROD activity and amount of CYP1A enzyme in haddock from Tampen (Fig. 3a,c). The EROD activity and CYP1A enzyme values were comparatively high even at the Egersund bank. For example, the EROD activity in haddock from Iceland was only 6% (13.6±10.4 pmol per min and mg protein, n = 20, not shown) of the EROD activity in haddock from the Egersund bank. This difference is too large to be explained only by normal seasonal variation. Moreover, in Atlantic cod from Lofoten, Norway, collected in the autumn 2000 and 2001, the EROD activity was below 10 pmol per min and mg protein [28]. This is less than 15% of the EROD activity in Atlantic cod from the Egersund bank. Our interpretation is that CYP1A is substrate induced even at the Egersund bank, and that the true background EROD activity of haddock and Atlantic cod should be lower.

Ideally, when the EROD activity is directly proportional to the amount of active CYP1A enzyme, the (species-specific) ratio between the two variables should be constant. In haddock this ratio was 1.2 nmol per min and absorbance at Tampen and 1.1 nmol per min and absorbance at the Egersund bank. The difference was not significant (Student's *t*-test, *p*>0.05, not shown). In Atlantic cod the ratio was 10.2 nmol per min and absorbance at Tampen and 6.9 nmol per min and absorbance at the Egersund bank. The difference was significant (Student's *t*-test, *p* = 0.025, not shown) and the two most likely explanations are either interaction between agonists in the complex mixture of petroleum pollutants, or contribution of other cytochrome P450 isoforms to the deethylation of ethoxyresorufin, in the Atlantic cod from Tampen.

**Figure 4:**
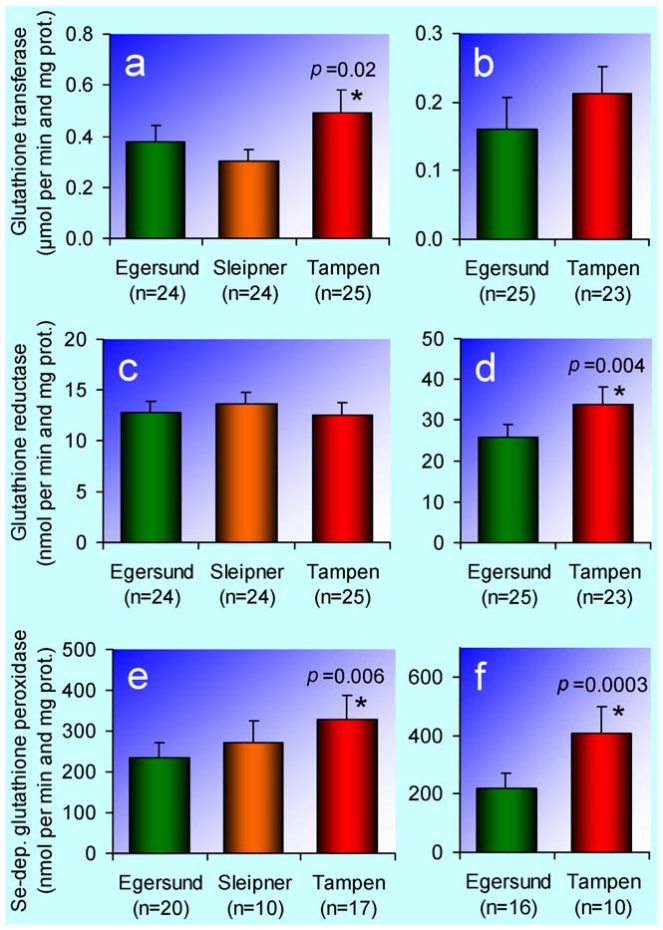
Hepatic glutathione dependent enzyme activities. **a**, Glutathione transferase (1-chloro-2,4-dinitrobenzene) in haddock (*Melanogrammus aeglefinus*). **b**, Glutathione transferase (1-chloro-2,4-dinitrobenzene) in Atlantic cod (*Gadus morhua*). **c**, Glutathione reductase in haddock. **d**, Glutathione reductase in Atlantic cod. **e**, Selenium-dependent glutathione peroxidase in haddock. **f**, Selenium-dependent glutathione peroxidase in Atlantic cod. The bars represent arithmetic means and the error bars 95% confidence intervals. An asterisk (*) indicates that the mean is significantly different (*p*<0.05) from the mean for the Egersund bank (control).

Glutathione transferases are a family of enzymes catalysing the conjugation of many PAHs and other xenobiotics with glutathione. In most vertebrates, the glutathione transferase activity measured with 1-chloro-2,4-dinitrobenzene as the second substrate probably reflects the overall activity of the glutathione transferase isoforms responsible for the conjugation of PAH metabolites. This is most likely true also for the haddock and the Atlantic cod. The glutathione transferase activity was elevated in haddock from Tampen (Fig. 4a), and there was a tendency towards a corresponding elevation in Atlantic cod from Tampen (Fig. 4b). Our interpretation is that also this group of biotransformation enzymes is substrate induced by petroleum pollutants.

The enzyme glutathione reductase is responsible for the maintenance of glutathione in the reduced form. Glutathione has several functions in the body. One of them is conjugation with metabolites of PAHs and other xenobiotics. Another important function of reduced glutathione is to act as an antioxidant. The glutathione reductase activity was elevated in Atlantic cod from Tampen (Fig. 4d). In haddock the glutathione reductase activity was similar at all three sites (Fig. 4c).

The enzyme selenium-dependent glutathione peroxidase is part of the antioxidant defence of the body. It functions as a highly efficient catalyst to reduce a wide variety of intracellular peroxides, including hydrogen and lipid peroxides, thereby detoxifying these potentially damaging molecules [29]. The selenium-dependent glutathione peroxidase activity was elevated in both haddock (Fig. 4e) and Atlantic cod (Fig. 4f) from Tampen, and in haddock there was a graded response with respect to the intensity of the oil production in the investigated areas (Fig. 4e). Our interpretation is that this enzyme is induced by oxidative stress caused by petroleum pollutants.

### Changes in fatty acid composition

**Figure 5:**
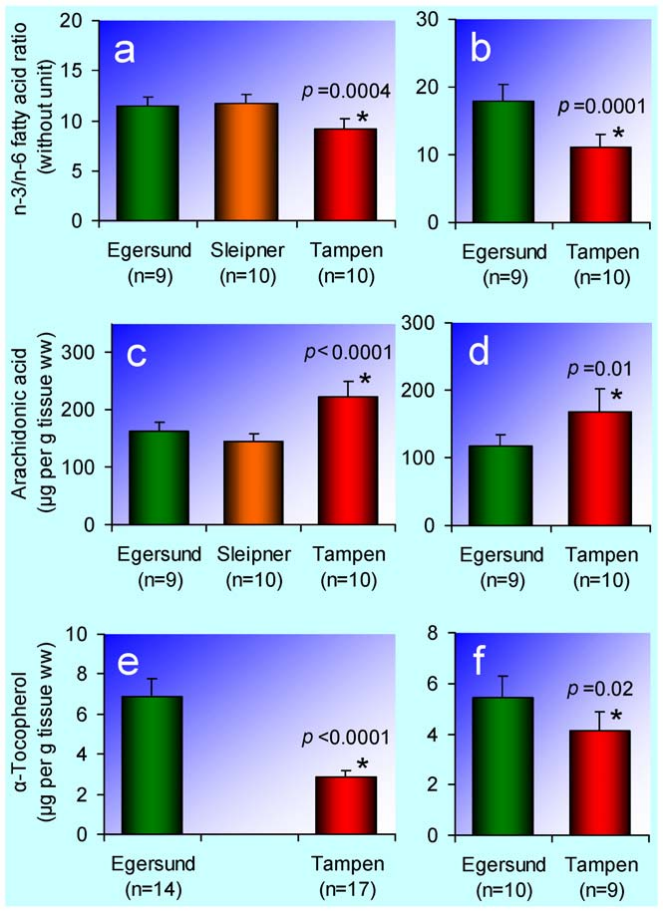
Fatty acids and α-tocopherol. **a**, The n-3 to n-6 fatty acid ratio in the muscle of haddock (*Melanogrammus aeglefinus*). **b**, The n-3 to n-6 fatty acid ratio in the muscle of Atlantic cod (*Gadus morhua*). **c**, Concentration of arachidonic acid in the liver (ww) of haddock. **d**, Concentration of arachidonic acid in the liver (ww) of Atlantic cod. **e**, Concentration of α-tocopherol in the muscle (ww) of haddock. **f**, Concentration of α-tocopherol in the muscle (ww) of Atlantic cod. The bars represent arithmetic means and the error bars 95% confidence intervals. An asterisk (*) indicates that the mean is significantly different (*p*<0.05) from the mean for the Egersund bank (control).

The ratio of n-3 to n-6 fatty acids in the muscle is both an indicator of the fatty acid composition and a measure of the nutritional value for human consumption. In Atlantic cod, the major part of the total muscle lipid is phospholipids [30], and the same would be expected for haddock. Accordingly, the ratio measured here reflects the fatty acid composition of the membranes. The muscle n-3 to n-6 fatty acid ratio was reduced in both haddock (Fig. 5a) and Atlantic cod (Fig. 5b) from Tampen. This result may be explained by a wide range of causes. In the living cell, the fatty acid composition of the membranes is highly regulated [31], [32]. Petroleum hydrocarbons may accumulate in the membranes, thereby altering their properties, or interfere directly with the metabolic reactions and/or molecular signalling regulating the fatty acid composition of the membranes. Specifically, reduced amount of n-3 fatty acids has been reported in Atlantic cod exposed in the laboratory to alkylphenols, which are present in relatively high concentrations in produced water [33]. Another possibility is that oxidative stress [34]–[36] alters the fatty acid composition of the membranes by lipid peroxidation [37]. Natural factors, like temperature and diet, are less likely to be responsible for the observed effects. The temperature at the Egersund bank was 9.3–17.6°C (average 16.4°C) at 10 m depth and 7.3–8.0°C (average 7.5°C) at the bottom, whereas the temperature at Tampen was 10.1–16.1°C (average 13.8°C) at 10 m depth and 8.3–10.1°C (average 9.0°C) at the bottom. These temperature differences are too small to give rise to any measurable change in fatty acid composition. In studies of the effect of temperature on membrane lipids in fish [e.g. 31,38,39], the investigated temperature differences were much larger. The extent of any dietary influence on the fatty acid composition in this investigation is unknown. Extreme diets in experiments with Atlantic cod have resulted in changes in the muscle n-3 to n-6 fatty acid ratio of the same magnitude as the difference between the Egersund bank and Tampen [40], [41]. Such extremes are, however, not to be expected in the natural diet of haddock and Atlantic cod in the investigated areas. Natural variation in the muscle n-3 to n-6 fatty acid ratio between populations that are reproductively isolated from each other cannot be excluded. Little is known, however, about such variation.

Arachidonic acid and many of its metabolites have multiple functions in cell signalling [42]–[44]. Arachidonic acid is also an important constituent of biological membranes [44]. Here, arachidonic acid was measured in the total liver lipid, which may constitute 30–76% of the haddock and Atlantic cod liver, depending on latitude and time of the year [45]. More than 90% of the total liver lipid may be triglycerides, and in comparison, the phospholipid proportion of the total liver lipid is negligible [46]. The concentration of arachidonic acid in the liver was elevated in both haddock (Fig. 5c) and Atlantic cod (Fig. 5d) from Tampen. These observations further support the hypothesis of altered fatty acid metabolism.

α-Tocopherol is one of eight lipid-soluble substances covered by the generic term vitamin E [47]. α-Tocopherol has antioxidant properties and may be consumed by reactive oxygen species [47], although other, and probably more important, functions *in vivo* are currently the subject of creative research efforts [48] and an animated debate [49]. The concentration of α-tocopherol in the muscle (and accordingly the nutritional value) was reduced in both haddock (Fig. 5e) and Atlantic cod (Fig. 5f) from Tampen. A possible explanation is depletion due to oxidative stress.

### Hepatic DNA adducts

DNA adducts are formed when reactive electrophilic metabolites of xenobiotics or endobiotics bind covalently to the DNA, which contains approximately 20 sites susceptible to adduct formation. Such reactive electrophilic metabolites also readily attack RNA, protein, and other cell constituents, thereby increasing the risk of a wide range of dysfunctions. Consequently, DNA adducts may be used both as a measure of the direct genotoxic effect, and as an indicator of a wider range of toxic exposure [50], [51]. *In vivo*, DNA adducts may give rise to a multitude of genotoxic effects – from immediate cell death to the development of cancer in subsequent generations [52]–[55].

The most sensitive method for DNA adduct analysis is the nuclease P1 version [20] of the ^32^P-postlabelling method [56], designed for the analysis of large hydrophobic aromatic adducts. DNA adducts, analysed in this way, have been widely used as a biomarker in fish [e.g. 18,19,57–59] and are considered to be one of the best biomarkers of PAH exposure. To our knowledge, DNA adducts are still the only biomarker of genotoxicity that does not require prior knowledge about the exact structure of the genotoxic substances. The application of two-dimensional TLC allows both fingerprinting (see [60] for an example) and quantification of the DNA adducts.

**Figure 6:**
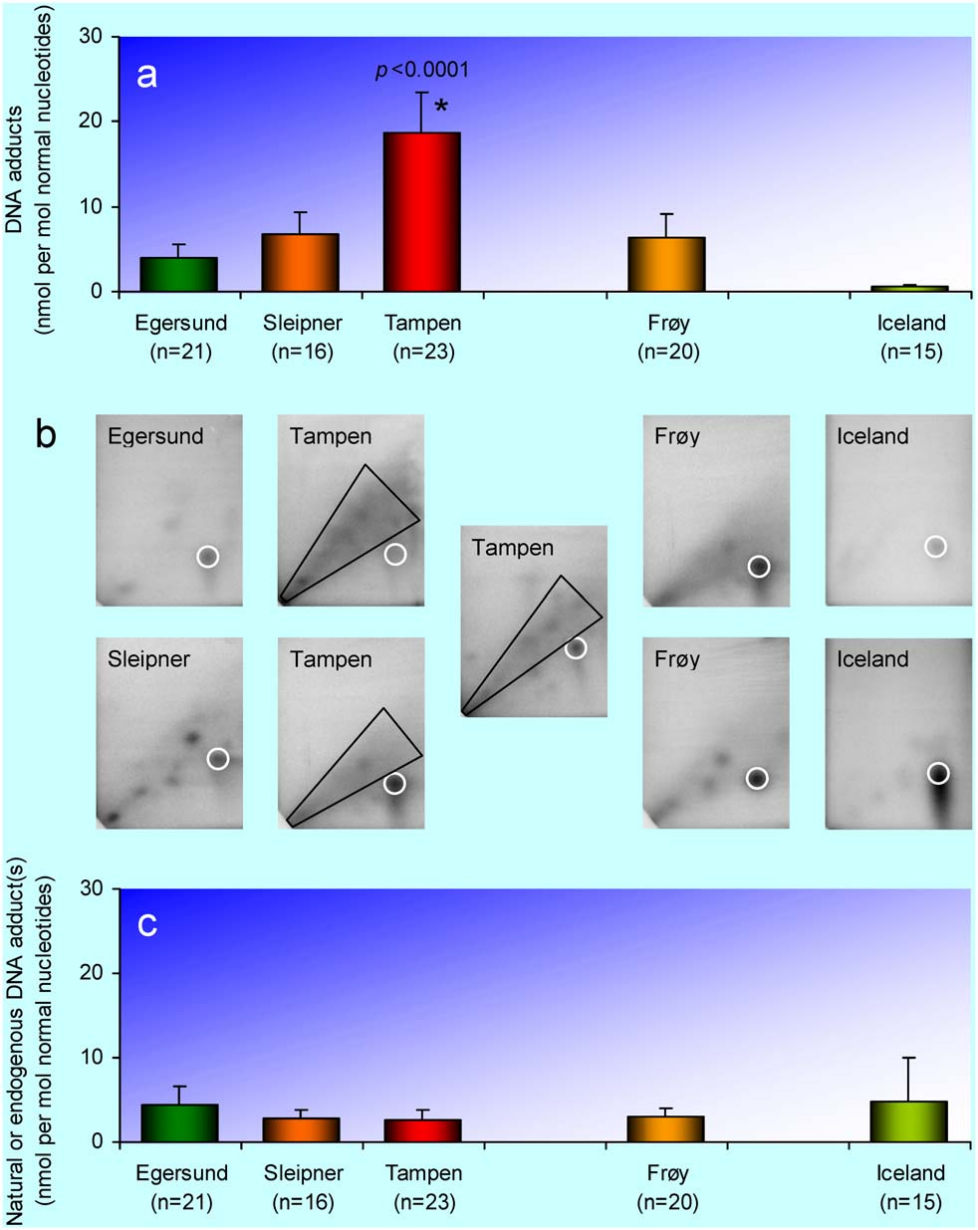
Hepatic DNA adducts. **a**, The level of DNA adducts in haddock (*Melanogrammus aeglefinus*) exclusive of the possible natural or endogenous DNA adduct(s). **b**, Representative autoradiograms for haddock from the investigated areas. The typical diagonal radioactive zone (DRZ) is indicated in the autoradiograms for specimens from Tampen. The possible natural or endogenous DNA adduct(s) is encircled in white. **c**, The level of the possible natural or endogenous DNA adduct(s) in haddock. The bars represent arithmetic means and the error bars 95% confidence intervals. An asterisk (*) indicates that the mean is significantly different (*p*<0.05) from the mean for the Egersund bank (control).

The level of hepatic DNA adducts in haddock from Tampen was considerably higher than at any other site (Fig. 6a). Intermediate levels were found at Sleipner and Frøy, and a relatively low level was found at the Egersund bank (Fig. 6a). It should be pointed out, however, that only the haddock from southwest of Iceland fulfilled the criterion for background level of hepatic DNA adducts (≤1.0±0.5 nmol adducts per mol normal nucleotides) [21]. Both Sleipner and Frøy, but not the Egersund bank, differed significantly from Iceland (Sleipner *p* = 0.007, Frøy *p* = 0.008, Egersund bank *p* = 0.104, not shown). The typical DNA adduct pattern was a diffuse diagonal radioactive zone (DRZ), indicative of a broad spectrum of DNA adducts (Fig. 6b). Similar DRZ patterns have been reported previously in other vertebrate species exposed to complex mixtures of PAHs from known and unknown sources [e.g. 57,61].

The very high DNA adduct levels at Tampen (∼20 nmol adducts per mol normal nucleotides) were similar to the DNA adduct levels found in perch (*Perca fluviatilis*) from contaminated areas measured with the same method. In the PAH polluted recipient of an aluminium smelter, the perch had DNA adduct levels of 29 nmol adducts per mol normal nucleotides [18], and in a creosote polluted area the perch had DNA adduct levels of 7 nmol adducts per mol normal nucleotides [60]. The DNA adduct levels at Tampen were also similar to the DNA adduct levels found in Atlantic cod exposed in the laboratory to 1 ppm dispersed crude oil for 3–16 days [25]. We propose that the very high level of DNA adducts at Tampen primarily is caused by the continuous discharge of produced water, although old drilling waste from previous operations may contribute, as indicated by the intermediate level of DNA adducts at the former oil production site Frøy.

In addition to the DRZ, we found a specific adduct spot, which occurred in every specimen (even in the Icelandic haddock) and was uncorrelated with the other adducts and the DRZ. This may be an example of a natural or endogenous DNA adduct, although such DNA adducts are very unusual. For example, no natural or endogenous DNA adducts were found in perch from a truly pristine area [60], in Northern pike (*Esox lucius*) from a breeding facility [19], or in 11 other fish species from the arctic or sub-arctic areas of the North Atlantic [21]. The natural or endogenous DNA adduct in haddock was excluded from the DNA adducts presented in Fig. 6a and is presented separately in Fig. 6c. The frequency of this adduct was similar at all sites (Fig. 6c). The adduct may originate from an endogenous substance, a natural toxin, or a widespread anthropogenic genotoxic substance. It could also reflect endogenous modification of a DNA base in the regulation of gene expression. Natural or endogenous DNA adducts occasionally found in mammals have been proposed to arise from ageing, normal dietary factors, or endogenous substances, such as steroids [62]. Further research should be performed to find the exact cause of this natural or endogenous DNA adduct in haddock.

### Effects of habitat and migration

The relatively high proportion of small PAHs (2- and 3-rings) in produced water may restrain the generation of DNA adducts by the larger PAHs (4-, 5- and 6-rings) [63], [64]. Such large PAHs bind particularly well to settling matter in the water column and are thus accumulated in the sediment at a higher rate than small PAHs. By measuring DNA adducts in haddock, which often feeds on the seafloor, we increased the probability to find DNA adducts formed by the larger PAHs. Models have indicated a high sedimentation rate in the three investigated areas in the North Sea [65]. This may explain why we find DNA adduct levels in the open sea that are similar to the DNA adduct levels in fish from highly polluted areas close to a point source.

Both haddock and Atlantic cod migrate seasonally [66]. Accordingly, they cannot be assumed to have resided in the investigated areas longer than since the spawning in the spring, although repeated exposure is possible if these species return to the same polluted area every year. Owing to the migration of haddock and Atlantic cod, the biological effects of petroleum pollution on more sedentary species may be even stronger. The haddock from Frøy was collected in the summer, a few months earlier than the haddock from the Egersund bank, Sleipner, and Tampen. Hence, there is a risk that the biomarker response at Frøy is underestimated, owing to a shorter period of exposure. For example, it took several months (between 16 and 44 weeks) to develop hepatic DNA adducts above the background in Atlantic cod exposed to a cocktail of low-molecular weight PAHs and short-chained alkylphenols in an experiment intended to mimic field exposure to produced water [67].

### Causes of the observed effects

It has been estimated that 2.9 million m^3^ of drill cuttings and muds, discharged from approximately 1300 wells, had accumulated on the Norwegian continental shelf by 2000 [68]. How much of this waste that still remains at the sites where it was formed is difficult to estimate. It is suspected, however, that a significant proportion has been spread by sea currents over a wider area in the North Sea and the North Atlantic. The investigated fish species are exposed to produced water, drill cuttings, muds, and accidental spills from the offshore oil production simultaneously. There are, of course, also other, more diffuse, pollution sources in the North Sea, but in comparison with the oil production in areas like Tampen and Sleipner, such pollution sources must be considered as minor. The results of this study do not give any detailed information about the relative contribution to the biomarker responses from the different major pollution sources. Neither can the most toxic substances be determined. The relationships between pollution sources, toxic substances and biomarker responses may also differ between areas and fish species.

### Conclusions

There was a general relationship between the intensity of oil production in the investigated North Sea areas and the biomarker responses in haddock and Atlantic cod. The biological effects included induction of biotransformation enzymes, oxidative stress, altered fatty acid composition, and genotoxicity. PAH metabolites were also demonstrated in the bile. It is most remarkable to obtain biomarker responses in natural fish populations in the open sea that are similar to the biomarker responses in fish from highly polluted areas close to a point source.

The results of the measurements of EROD activity and DNA adducts raise the question whether the Egersund bank is a valid control. If Sleipner and Tampen are instead compared with the waters southwest of Iceland, the biological effects of oil production appear to be much stronger. Hence, the validity of the Egersund bank, or any other area in the North Sea, as control requires further investigation.

Risk assessment of various threats to the marine fish populations in the North Sea, such as overfishing, global warming [69], and eutrophication [70], should also take into account the ecologically relevant impact of offshore oil production.

## Supporting Information

Dataset S1 Raw data on which the statistical analyses are based.
(XLS)

## References

[1] Neff JM (2002). Bioaccumulation in marine organisms: effect of contaminants from oil well produced water..

[2] Anonymous (2003). Oil in the Sea III – inputs, fates and effects..

[3] Hylland K, Beyer J, Berntssen M, Klungsøyr J, Lang T (2006). May persistent organic pollutants affect fish populations in the North Sea?. J Toxicol Environ Health, A.

[4] Aas E, Beyer J, Goksøyr A (2000). Fixed wavelength fluorescence (FF) of bile as a monitoring tool for polyaromatic hydrocarbon exposure in fish: an evaluation of compound specificity, inner filter effect and signal interpretation.. Biomarkers.

[5] Beyer J, Jonsson G, Porte C, Krahn MM, Ariese F (2010). Analytical methods for determining metabolites of polycyclic aromatic hydrocarbon (PAH) pollutants in fish bile: A review.. Environ Toxicol Pharmacol.

[6] Jonsson G, Beyer J, Wells D, Ariese F (2003). The application of HPLC-F and GC-MS to the analysis of selected hydroxy polycyclic hydrocarbons in two certified fish bile reference materials.. J Environ Monit.

[7] Burke MD, Mayer RT (1974). Ethoxyresorufin: Direct fluorimetric assay of a microsomal O-dealkylation which is preferentially inducible by 3-methylcholanthrene.. Drug Metab Dispos.

[8] Goksøyr A, Larsen HE, Husøy A (1991). Application of a cytochrome P-450 IA1-ELISA in environmental monitoring and toxicological testing of fish.. Comp Biochem Physiol.

[9] Habig WH, Pabst MJ, Jakoby WB (1974). Glutathione S-transferase, the first enzymatic step in mercapturic acid formation.. J Biol Chem.

[10] Cribb AE, Leeder JS, Spielberg SP (1989). Use of microplate reader in an assay of glutathione reductase using 5,5′-dithiobis(2-nitrobenzoic acid).. Anal Biochem.

[11] Bell JG, Cowey CB, Adron JW, Shanks AM (1985). Some effects of vitamin E and selenium deprivation on tissue enzyme levels and indices of tissue peroxidation in rainbow trout (*Salmo gairdneri*).. Br J Nutr.

[12] Lowry OH, Rosebrough NJ, Farr AL, Randall RJ (1951). Protein measurement with the Folin phenol reagent.. J Biol Chem.

[13] Torstensen BE, Frøyland L, Lie Ø (2004). Replacing dietary fish oil with increasing levels of rapeseed oil and olive oil – effects on Atlantic salmon (*Salmo salar* L.) tissue and lipoprotein lipid composition and lipogenic enzyme activities.. Aquacult Nutr.

[14] Lie Ø, Sandvin A, Waagbø R (1994). Transport of tocopherol in Atlantic salmon (*Salmo salar*) during vitellogenesis.. Fish Physiol Biochem.

[15] Dunn BP, Black JJ, Maccubbin A (1987). ^32^P-Postlabeling analysis of aromatic DNA adducts in fish from polluted areas.. Cancer Res.

[16] Beach AC, Gupta RC (1992). Human biomonitoring and the ^32^P-postlabeling assay.. Carcinogenesis.

[17] Reichert WL, French B (1994). The ^32^P-postlabeling protocols for assaying levels of hydrophobic DNA adducts in fish. NOAA Technical Memorandum NMFS-NWFSC-14..

[18] Ericson G, Lindesjöö E, Balk L (1998). DNA adducts and histopathological lesions in perch (*Perca fluviatilis*) and northern pike (*Esox lucius*) along a polycyclic aromatic hydrocarbon gradient on the Swedish coastline of the Baltic Sea.. Can J Fish Aquat Sci.

[19] Ericson G, Balk L (2000). DNA adduct formation in northern pike (*Esox lucius*) exposed to a mixture of benzo[a]pyrene, benzo[k]fluoranthene and 7*H*-dibenzo[c,g]carbazole: time-course and dose-response studies.. Mutat Res, Fundam Mol Mech Mutagen.

[20] Reddy MV, Randerath K (1986). Nuclease P1-mediated enhancement of sensitivity of ^32^P-postlabeling test for structurally diverse DNA adducts.. Carcinogenesis.

[21] Aas E, Liewenborg B, Grøsvik BE, Camus L, Jonsson G (2003). DNA adduct levels in fish from pristine areas are not detectable or low when analysed using the nuclease P1 version of the ^32^P-postlabelling technique.. Biomarkers.

[22] Leach JJ, Pepple SK, Statham CN (1973). Fish bile analysis: a possible aid in monitoring water quality.. Toxicol Appl Pharmacol.

[23] Balk L, Meijer J, DePierre JW, Appelgren L-E (1984). The uptake and distribution of [^3^H]benzo(a)pyrene in the Northern pike (*Esox lucius*). Examination by whole-body autoradiography and scintillation counting.. Toxicol Appl Pharmacol.

[24] Varanasi U, Stein JE, Nishimoto M, Varanasi U (1989). Biotransformation and disposition of PAH in fish.. Metabolism of polycyclic aromatic hydrocarbons in the aquatic environment. CRC Uniscience Series.

[25] Aas E, Baussant T, Balk L, Liewenborg B, Andersen OK (2000). PAH metabolites in bile, cytochrome P4501A and DNA adducts as environmental risk parameters for chronic oil exposure: A laboratory experiment with Atlantic cod.. Aquat Toxicol.

[26] Grung M, Holth TF, Rindal Jacobsen M, Hylland K (2009). Polycyclic aromatic hydrocarbon (PAH) metabolites in Atlantic cod exposed via water or diet to a synthetic produced water.. J Toxicol Environ Health, Part A.

[27] Sundt RC, Meier S, Jonsson G, Sanni S, Beyer J (2009). Development of a laboratory exposure system using marine fish to carry out realistic effect studies with produced water discharged from offshore oil production.. Mar Pollut Bull.

[28] Ruus A, Hylland K, Green NW (2003). Joint Assessment and Monitoring Programme (JAMP)..

[29] Gamble SC, Wiseman A, Goldfarb PS (1997). Selenium-dependent glutathione peroxidase and other selenoproteins: their synthesis and biochemical roles.. J Chem Technol Biotechnol.

[30] Exler J, Kinsella JE, Watt BK (1975). Lipids and fatty acids of important finfish: new data for nutrient tables.. J Am Oil Chem Soc.

[31] Farkas T, Fodor E, Kitajka K, Halver JE (2001). Response of fish membranes to environmental temperature.. Aquacult Res.

[32] Rilfors L, Lindblom G (2002). Regulation of lipid composition in biological membranes – biophysical studies of lipids and lipid synthesizing enzymes.. Colloids Surf, B.

[33] Meier S, Andersen TC, Lind-Larsen K, Svardal A, Holmsen H (2007). Effects of alkylphenols on glycerophospholipids and cholesterol in liver and brain from female Atlantic cod (*Gadus morhua*).. Comp Biochem Physiol, Part C.

[34] Sturve J, Hasselberg L, Fälth H, Celander M, Förlin L (2006). Effects of North Sea oil and alkylphenols on biomarker responses in juvenile Atlantic cod (*Gadus morhua*).. Aquat Toxicol.

[35] Bohne-Kjersem A, Skadsheim A, Goksøyr A, Grøsvik BE (2009). Candidate biomarker discovery in plasma of juvenile cod (*Gadus morhua*) exposed to crude North Sea oil, alkyl phenols and polycyclic aromatic hydrocarbons (PAHs).. Mar Environ Res.

[36] Farmen E, Harman C, Hylland K, Tollefsen K-E (2010). Produced water extracts from North Sea oil production platforms result in cellular oxidative stress in a rainbow trout in vitro bioassay.. Mar Pollut Bull.

[37] Josephy PD, Mannervik B, Ortiz de Montellano P (1997). Chapter 7. Lipid peroxidation.. Molecular toxicology.

[38] Dey I, Buda C, Wiik T, Halver JE, Farkas T (1993). Molecular and structural composition of phospholipid membranes in livers of marine and freshwater fish in relation to temperature.. Proc Natl Acad Sci USA.

[39] Grim JM, Miles DRB, Crockett EL (2010). Temperature acclimation alters oxidative capacities and composition of membrane lipids without influencing activities of enzymatic antioxidants or susceptibility to lipid peroxidation in fish muscle.. J Exp Biol.

[40] Lie Ø, Lied E, Lambertsen G (1986). Liver retention of fat and of fatty acids in cod (*Gadus morhua*) fed different oils.. Aquaculture.

[41] Santos J, Burkow IC, Jobling M (1993). Patterns of growth and lipid deposition in cod (*Gadus morhua* L.) fed natural prey and fish-based feeds.. Aquaculture.

[42] Needleman P, Turk J, Jakschik BA, Morrison AR, Lefkowith JB (1986). Arachidonic acid metabolism.. Annu Rev Biochem.

[43] Piomelli D (1993). Arachidonic acid in cell signalling.. Curr Opin Cell Biol.

[44] Brash AR (2001). Arachidonic acid as a bioactive molecule.. J Clin Invest.

[45] Falch E, Rustad T, Jonsdottir R, Shaw NB, Dumay J (2006). Geographical and seasonal differences in lipid composition and relative weight of by-products from gadiform species.. J Food Compos Anal.

[46] Falch E, Rustad T, Aursand M (2006). By-products from gadiform species as raw material for production of marine lipids as ingredients in food or feed.. Process Biochem.

[47] Burton GW (1994). Vitamin E: molecular and biological function.. Proc Nutr Soc.

[48] Brigelius-Flohé R, Galli F (2010). Vitamin E: A vitamin still awaiting the detection of its biological function.. Mol Nutr Food Res.

[49] Brigelius-Flohé R (2010). Widened horizon of vitamin E research.. Mol Nutr Food Res.

[50] Kurelec B (1993). The genetic disease syndrome.. Mar Environ Res.

[51] Shugart LR, Rand GM (1995). Environmental genotoxicology.. Aquatic toxicology. Effects, environmental fate, and risk assessment.

[52] Kirkwood TBL (1989). DNA, mutations and aging.. Mutat Res.

[53] Bridges BA, Bowyer DE, Hansen ES, Penn A, Wakabayashi K (1990). The possible involvement of somatic mutations in the development of atherosclerotic plaques. Report of ICPEMC Subcommittee 7/1. Conclusions and recommendations.. Mutat Res.

[54] Würgler FE, Kramers PGN (1992). Environmental effects of genotoxins (ecogenotoxicology).. Mutagenesis.

[55] Farmer PB, Shuker DEG (1999). What is the significance of increases in background levels of carcinogen-derived protein and DNA adducts? Some considerations for incremental risk assessment.. Mutat Res, Fundam Mol Mech Mutagen.

[56] Randerath K, Reddy MV, Gupta RC (1981). ^32^P-labeling test for DNA damage.. Proc Natl Acad Sci USA.

[57] Varanasi U, Reichert WL, Stein JE (1989). ^32^P-postlabeling analysis of DNA adducts in liver of wild English sole (*Parophrys vetulus*) and winter flounder (*Pseudopleuronectes americanus*).. Cancer Res.

[58] Stein JE, Collier TK, Reichert WL, Casillas E, Hom T (1993). Bioindicators of contaminant exposure and sublethal effects in benthic fish from Puget Sound, WA, USA.. Mar Environ Res.

[59] Ericson G, Noaksson E, Balk L (1999). DNA adduct formation and persistence in liver and extrahepatic tissues of northern pike (*Esox lucius*) following oral exposure to benzo[*a*]pyrene, benzo[*k*]fluoranthene and 7H-dibenzo[*c*,*g*]carbazole.. Mutat Res, Fundam Mol Mech Mutagen.

[60] Ericson G, Liewenborg B, Lindesjöö E, Näf C, Balk L (1999). DNA adducts in perch (*Perca fluviatilis*) from a creosote contaminated site in the River Ångermanälven, Sweden.. Aquat Toxicol.

[61] French BL, Reichert WL, Hom T, Nishimoto M, Sanborn HR (1996). Accumulation and dose-response of hepatic DNA adducts in English sole (*Pleuronectes vetulus*) exposed to a gradient of contaminated sediments.. Aquat Toxicol.

[62] Liehr JG, Avitts TA, Randerath E, Randerath K (1986). Estrogen-induced endogenous DNA adduction: Possible mechanism of hormonal cancer.. Proc Natl Acad Sci USA.

[63] McKee MJ, Hendricks AC, Ebel RE (1983). Effects of naphthalene on benzo[a]pyrene hydroxylase and cytochrome P-450 in *Fundulus heteroclitus*.. Aquat Toxicol.

[64] Willett KL, Wassenberg D, Lienesch L, Reichert W, Di Giulio RT (2001). *In vivo* and *in vitro* inhibition of CYP1A-dependent activity in *Fundulus heteroclitus* by the polynuclear aromatic hydrocarbon fluoranthene.. Toxicol Appl Pharmacol.

[65] Stagg RM, Gillibrand PA, McIntosh AM, Turrell WA, Reed M, Johnsen S (1996). The effect of produced water on hydrocarbon levels and on P4501A monooxygenase activity in fish larvae in the northern North Sea.. Produced water 2: environmental issues and mitigation technologies.

[66] Knijn RJ, Boon TW, Heessen HJL, Hislop JRG (1993). Atlas of North Sea Fishes. ICES Cooperative Research Report No. 194..

[67] Holth TF, Beylich BA, Skarphédinsdóttir H, Liewenborg B, Grung M (2009). Genotoxicity of environmentally relevant concentrations of water-soluble oil components in cod (*Gadus morhua*).. Environ Sci Technol.

[68] Anonymous (2001). Survey of information on cutting piles in the Norwegian sector. Technical report..

[69] O'Brien CM, Fox CJ, Planque B, Casey J (2000). Climate variability and North Sea cod.. Nature.

[70] Diaz RJ, Rosenberg R (2008). Spreading dead zones and consequences for marine ecosystems.. Science.

